# Rainforest Pharmacopeia in Madagascar Provides High Value for Current Local and Prospective Global Uses

**DOI:** 10.1371/journal.pone.0041221

**Published:** 2012-07-27

**Authors:** Christopher D. Golden, B. J. Rodolph Rasolofoniaina, E. J. Gasta Anjaranirina, Lilien Nicolas, Laurent Ravaoliny, Claire Kremen

**Affiliations:** 1 Harvard University Center for the Environment, Cambridge, Massachusetts, United States of America; 2 Madagascar Health and Environmental Research (MAHERY), Maroantsetra, Madagascar; 3 Department of Environmental Science Policy and Management, University of California, Berkeley, California, United States of America; 4 Maroantsetra District Public Hospital, Maroantsetra, Madagascar; University of Arkansas, United States of America

## Abstract

Botanical diversity provides value to humans through carbon sequestration, air and water purification, and the provisioning of wild foods and ethnomedicines. Here we calculate the value of botanical ethnomedicines in a rainforest region of Madagascar, the Makira Protected Area, using a substitution method that combines replacement costs and choice modeling. The Makira watershed may comprise approximately 0.8% of global botanical diversity and possesses enormous value both in its ability to provision botanical ethnomedicines to local people and as a source of potentially novel pharmaceutical drugs for society as a whole. Approximately 241 locally-recognized species are used as ethnomedicines, including 113 agricultural or weed species. We equated each ethnomedicinal treatment to the monetary value of a comparable pharmaceutical treatment adjusted by personal preferences in perceived efficacy (rather than from known or assumed medicinal equivalency). The benefit value of these botanical ethnomedicines per individual is $5.40–7.90 per year when using the value of highly subsidized Malagasy pharmaceuticals and $100.60–287.40 when using the value of American pharmaceuticals. Using local pharmaceuticals as substitutes, the value per household is $30.24–44.30 per year, equivalent to 43–63% of median annual household income, demonstrating their local importance. Using the value of American pharmaceuticals, the amount is equivalent to 22–63% of the median annual health care expenditures for American adults under 45 in 2006. The potential for developing novel biomedicines from the Makira watershed’s unique flora ranges in untapped benefit value from $0.3–5.7 billion for American pharmaceutical companies, non-inclusive of the importance of providing novel medicines and improved healthcare to society. This study provides evidence of the tremendous current local and prospective global value of botanical ethnomedicines and furthers arguments for the conservation of tropical forests for sustainable use.

Botanique de la diversité apporte de la valeur à l’homme par la séquestration du carbone, de l’air et de purification de l’eau, et le provisionnement des aliments sauvages et ethnomedicines. Ici, nous calculons la valeur de ethnomedicines botaniques dans une région de forêt de Madagascar, la zone protégée de Makira, en utilisant une méthode de substitution qui combine les coûts de remplacement et la modélisation des choix. Le bassin versant de Makira peut comprendre environ 0,8% de la diversité botanique mondiale et possède une valeur énorme à la fois dans sa capacité à fournir ethnomedicines botaniques à la population locale et en tant que source de nouveaux médicaments potentiellement pharmaceutiques pour la société dans son ensemble. Environ 241 espèces localement reconnus sont utilisés comme ethnomedicines, y compris 113 espèces d’agricoles ou de mauvaises herbes. Nous assimilé chaque traitement ethnomédicales à la valeur monétaire d’un traitement comparable pharmaceutique ajusté en fonction des préférences personnelles en matière d’efficacité perçue (plutôt que de l’équivalence médicament connu ou supposé). La valeur de l’avantage de ces ethnomedicines botaniques par individu est de $5,40 à 7.90 par année lors de l’utilisation de la valeur des produits pharmaceutiques malgaches fortement subventionnés et de $100,60 à 287,40 lors de l’utilisation de la valeur des produits pharmaceutiques américains. Utilisation de produits pharmaceutiques locales comme des substituts, la valeur par ménage est de $30.24 à 44.30 par an, équivalent à 43–63% du revenu médian des ménages annuelle, ce qui démontre leur importance locale. Utilisation de la valeur des produits pharmaceutiques américaines, le montant est équivalent à 22–63% de la médiane des dépenses annuelles de soins de santé pour les adultes américains de moins de 45 en 2006. Le potentiel de développement de nouveaux biomédicaments des fourneaux dans le bassin versant de la flore Makira unqiue de la valeur des avantages inexploité de $0,3 à 5,7 milliards pour les sociétés pharmaceutiques américaines, non compris l’importance de fournir de nouveaux médicaments et de soins de santé amélioré à la société. Cette étude fournit une preuve de l’énorme valeur actuelle globale locale et prospective de ethnomedicines botaniques et des arguments fait avancer pour la conservation des forêts tropicales pour l’utilisation durable.

## Introduction

An estimated 52,885 plant species are used globally as medicines [1], approximately 1/6^th^ of all global botanical diversity [2]. Ethnomedicines provide a valuable resource and can be considered a provisioning ecosystem service (sensu [3]). These medicines are often utilized by local people living in developing countries, and are frequently the primary defense against illness, either due to cultural preference or lack of other formalized healthcare alternatives [4]. Historically, this primary defense against ill-health has been undervalued because, among other reasons, ethnomedicines are not market-integrated [3]. Ecosystem service valuations are useful to conservation and development practitioners for calculating the total value of benefits from ecosystems, choosing between alternative land use and management scenarios, ascertaining the distribution of the costs and benefits of services to users, and identifying or developing financing mechanisms for ecosystem services [5]. In this research, we illuminate the provisioning service value of botanical ethnomedicines by equating these traditional treatments to the use of pharmaceutical treatments for the same condition. Valuing ethnomedicines provides important information to the nation of Madagascar, demonstrating the value of forests for public health, and to the global commons, showing the potential for development of novel biomedicines.

Botanical ethnomedicines underpin healthcare for many resource-dependent cultures throughout the developing world. Deforestation or strict conservation management may cause the loss of access to these botanical ethnomedicines, causing increased reliance on Western biomedicines, and attendant costs to individuals and nations, which, in the developing world, frequently subsidize Western medicines [6]. Monetary valuations for non-market services, while difficult to estimate, can be calculated either through contingent valuation methods or through the substitution of a close proxy [5]. In Madagascar, there are approximately 3,500 types of botanical ethnomedicines [7], 6.6% of the estimated global total. Although widely used and well-understood by local people, this primary form of health care is often ignored and marginalized by promoters of Western biomedicine. Nevertheless, of 1,184 new FDA approved chemical entities, 70% have a biological origin [8].

The Malagasy’s use of ethnomedicines is not formalized into a traditional system of medicine with codified pharmacopeias (like Ayurvedic or Chinese ethnomedicine) but is transmitted by oral means and learned through participatory approaches. The majority of medicinal treatments fall into this category but a small fraction of treatments are reserved for the truly specialized spiritual healer, called *ombiasa*. The repertoire of medicines found in Madagascar is highly complex with a diverse range of species and treatment types. Providing a detailed monetary valuation of this ecosystem provisioning service will lend perspective to public health specialists, conservation planners, natural resource managers, and development agencies regarding the local importance of this service. Here we compare the value of this service to potential bioprospecting revenue and the UN-sponsored REDD (Reduced Emissions from Deforestation and Degradation) program to better understand the latent value of this ethnoknowledge and provide further evidence for supporting tropical forest conservation.

## Methods

### Sampling and Surveys

Through a process of systematic random sampling, we identified 634 households in 24 villages from 2005 until 2010 in the Makira Protected Area (MPA). The MPA is a lowland to mid-altitude rainforest in northeastern Madagascar, characterized by high levels of biodiversity [9]. Communities were selected by attempting to follow trade routes and travel passages to maximize the geographical and cultural variation of this landscape. Households were selected in each community by obtaining a full census, assigning a number to each household and then randomizing these numbers for selection. We attempted to enroll thirty households per community but were limited by community size on occasion. Our team used open-ended survey methodology to ask questions regarding the frequency of use and either the time allocated in searching for these particular botanicals or money spent in purchasing them. We also asked questions regarding the preparation, doses, and indicated treatments for each botanical ethnomedicine. We calculated the frequency of each household collecting any type of ethnomedicine and created a conservative estimate of user frequency by assuming only one user per collection per household. This is likely an underestimate because many members of a household will consume a prepared medicine if it is a generic treatment (i.e. a medicine treating fatigue rather than a medicine inducing labor). To account for limited sampling effort, we also used these data to calculate the total expected richness of medicinal plants with EstimateS [10] using both a non-parametric first-order jackknife and Chao’s non-parametric richness estimators [11], [12]. These methods are typically used for ecological surveys to adjust species richness counts for undetected species. Here, we used these methods to adjust for gaps in responses in social surveys.

Within these households, we interviewed the male and female head of households regarding the use of botanicals as medicines and whether these were appropriate for their children’s use as well. The individual who prepares ethnomedicines is almost exclusively the male or female head of household. Participant-observation methods were used to follow individuals into the forest to collect botanical ethnomedicines and observe them preparing them once returning to the village. Ethnographic methods were used to understand the meaning of particular illness categories, especially those that did not have a clear Western medical counterpart.

Ethnomedicinal data were categorized post-facto into treatments for 82 locally-recognized Malagasy illnesses. Malagasy translations for each treatment type can be found in Table S1. For the purposes of presentation clarity, treatments were categorized into frequent and infrequent treatments. All treatments of illnesses with less than 10 reported uses were presented separately and appear as “Other” in the main table of frequently used botanical treatments. Botanical ethnomedicines for diarrhea and bloody diarrhea were categorized jointly as one illness treatment. For illnesses with variations in regional dialectical naming, the most commonly used term was listed and the regional variants are listed (Table S2).

### Valuation Methods and Stated Preferences of Treatments

For the ethnomedicinal treatment for each of the 82 illness types, we identified the Western medicine counterpart used in Madagascar to treat the same ailment. Values were determined as the average value of a course of treatment using a comparable pill, syrup, or injection available at the pharmacy in the nearby town of Maroantsetra. Frequency of ethnomedicinal use (collected through oral recall of use during a prior week or prior month) was extrapolated to produce annual frequency estimates. The extrapolated frequency of ethnomedicine use was then multiplied by the average value of the corresponding course of Western pharmaceutical treatment for that illness. This value was weighted by the percent of the population sampled who preferred ethnomedicines over a given pharmaceutical treatment for each illness type. An exchange rate of 2,000 Ariary to $1USD was used (the current exchange rate in September 2011). All values were extrapolated to the entire population of the Makira Protected Area. With approximately 5.6 individuals per household (unpublished data), the estimated 140,000 people living in the MPA likely comprise 25,000 households.

Benefit (B) was then calculated as the total monetary benefits accrued over all ethnomedicines without subtracting the costs of time allocated in resource collection:


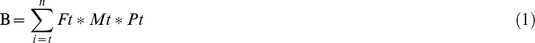


where *F* is the frequency of use of a given ethnomedicinal treatment *t* each year throughout the Makira Protected Area and *M* is the price of the Western biomedicine equivalent for a particular treatment type and *P* is the weight assigned to the price of the pharmaceutical based on the percentage of individuals preferring the ethnomedicinal treatment. Frequency is sensitive to the time in which the survey was given because there are certainly seasonal components to some diseases, such as malaria. With that said, we do not believe that our sampling strategy was heavily biased by this because households were surveyed throughout all twelve months of a year and over the course of five years. In this study, we use two different values for the price of the Western biomedicine (*M*): the local highly subsidized cost of treatment in Madagascar and the price of American online pharmaceuticals. The price for American online pharmaceuticals was recorded from www.drugstore.com. We use the price of American online pharmaceuticals as a point of comparison as pharmaceuticals are very highly subsidized in Madagascar and thus can be seen as a lower bound estimate of the value, whereas the price of American online pharmaceuticals can be seen as an upper bound. By providing both values, we are creating a confidence interval, in a sense. In a set of three villages in the MPA, we set out to investigate local people’s stated preferences of using Western pharmaceuticals or ethnobotanical medicines to treat a set of self-identified illnesses.

We surveyed 207 individuals (63 from villages with a distant hospital and 144 from a village with a pharmacy). Respondents were asked to report whether they preferred one type of medicine to the other given a particular illness and were allowed non-responses if a preference could not be determined. This question was phrased as a preference between the efficacy of each respective medicine ignoring price or distance needed to travel to obtain the pharmaceutical. Upper and lower ranges of value were determined for ethnomedicines by each illness type by weighting the price of the pharmaceutical by the percentage of respondents who preferred the ethnomedicinal treatment. The weight for the upper range was calculated as the percentage of respondents from a village with distant access to a pharmacy that preferred the perceived efficacy of the ethnomedicinal treatment and the weight for the lower range was the percentage of respondents living in a village with a hospital who preferred the perceived efficacy of the ethnomedicine. Thus, the values in this research are derived as personal preferences in perceived efficacy rather than from assumptions of medicinal equivalency. In addition to geographic proximity to available pharmaceuticals, there are also cultural mechanisms that could influence these preferences such as age (the younger generation may prefer Western biomedicines) or socio-economic status (the wealthy may avoid ethnomedicines as a reflection of poverty). We were not able to study these cultural mechanisms empirically but expect that age and wealth have not skewed our results.

### Cost Evaluation

In one village that was chosen specifically because it has the greatest number of plant types listed for medicinal use, we collected data on the approximate time traveled to collect ethnomedicines. This data was used to produce a maximum estimate of the area surrounding a village in which these ethnomedicines are harvested, assuming that 5 km could be traveled in one hour. The cost of time allocated to collecting ethnomedicines throughout the Makira was calculated by multiplying the following factors together across all households (*h*): the frequency of utilization (F), the median time allocated to collection (T), and the hourly wage rate of labor (W):


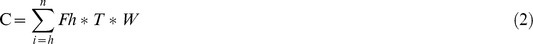


If a household does not use ethnomedicines, this is accounted for by having a frequency of zero. We do not have data for each household on time allocation and wage labor rate and thus use the median reported from our surveys. The median time allocated to collection included the round trip time spent accessing the ethnomedicine and the time spent harvesting, but did not include the time preparing it as this was often subsumed within meal preparation. An average of 5 minutes spent harvesting was estimated. The estimated hourly wage rate is $0.06–0.12 per hour (Golden, unpublished data) and we used the midpoint ($0.09 per hour) for this analysis. The net value of botanical ethnomedicine provisioning was calculated as the potential benefit accrued for ethnomedicine collection and use (Equation 1) minus the costs of time allocation (Equation 2).

### Ethics Statement

This study was approved by the University of California, Berkeley’s Committee on the Protection of Human Subjects (CPHS#2007-2–3), the Ministry of Water and Forests in Madagascar (#135/09/MEFT/SG/DGEF/DSAP/SLRSE) and the Maroantsetra District Hospital’s Medical Inspector. We also obtained approval from the listed boards to receive oral informed consent from all study participants because there are illiterate members of the population for whom reading consent documents and signing their name would be impossible.

## Results

**Figure 1:**
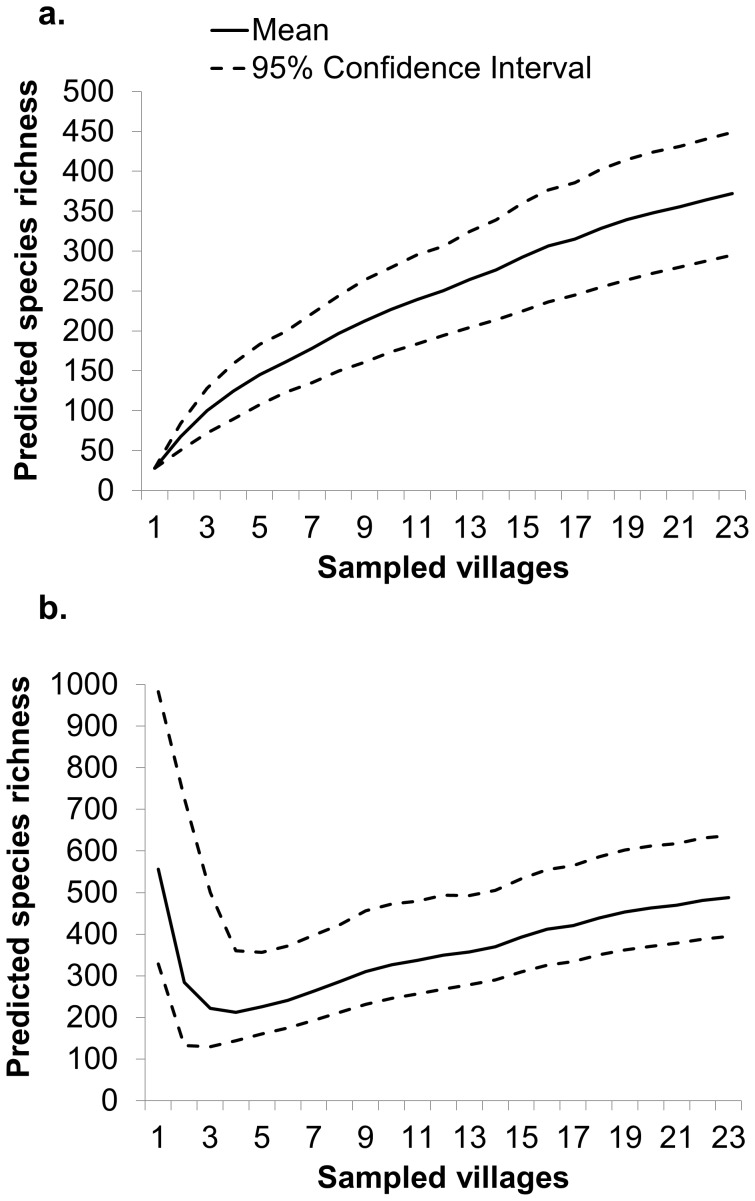
Potential number of ethnomedicines using a non-parametric jackknife richness estimator and Chao’s species richness estimator. As we were unable to sample the entire human population of the Makira Protected Area, we attempted to estimate the potential number of ethnomedicines within the forest by calculating a) a non-parametric first-order jackknife and b) Chao’s non-parametric richness estimators [11], [12].

Thirty-seven of 634 households (5.8%) reported no use of ethnomedicines and a complete reliance on Western pharmaceuticals to treat all illnesses. Of the remaining 94.2% (1,659 household-reports of ethnomedicine use), only 0.4% of ethnomedicines were purchased as opposed to collected. Only 7% of these household-reports evidenced a need to go a significant distance into the forest to collect botanical ethnomedcines, and the remainder stated that botanical ethnomedicines were collected nearby the village, within agricultural fields, or along paths to access farmland. Eighty-two categories of illness treated by ethnomedicines were observed in residents of the Makira Protected Area. Some botanical ethnomedicines were far more commonly used than others (Table 1). Two hundred and forty-one locally recognized plant species were used as treatments for the 82 categories of illness. Using both a non-parametric first-order jackknife and Chao’s non-parametric richness estimators, we estimated the likely richness of ethnomedicines to be between 372 (95% CI: 295–449) - 488 (95% CI: 395–637) respectively (Fig. 1a, b). Forty-seven different types of pharmaceutical medicines were employed to treat the same illnesses.

**Table 1:** The frequency of often-used botanical ethnomedicines.

Treatment Type	Percent	Preference for ethnomedicine(distant hospital)	Preference for ethnomedicine(on-site hospital)
Medicine for fatigue and muscle soreness	24.27%	70%	58%
Medicine for fatigue and dehydration	17.36%	76%	66%
Medicine for lower back and hip pain	11.75%	57%	49%
Stomach medicine	10.43%	68%	22%
Other	5.95%		
Fever medicine	5.21%	26%	6%
Medicine for muscle fatigue	4.81%	67%	32%
Medicine for back pain	4.66%	50%	31%
Medicine to increase strength	2.17%	74%	14%
Malaria medicine	2.13%	16%	8%
Medicine for enlarged testicles (also can mean a medicine to cure bedwetting)	1.10%	58%	34%
Medicine for headaches	1.06%	0%	1%
Medicine for anemia	0.99%	49%	21%
Medicine for gastro-intestinal ailments	0.88%	46%	31%
Medicine for dizziness or vertigo	0.81%	34%	17%
Medicine to cure tiredness	0.73%	68%	21%
Medicine for painful wisdom teeth	0.70%	19%	4%
Cough medicine	0.66%	0%	4%
Medicine to treat jaundice	0.59%	33%	12%
Medicine for toothaches	0.55%	23%	8%
Diarrhea medicine	0.48%	29%	11%
Medicine to treat genital sores and ulcers	0.44%	22%	4%
Medicine for warming the stomach (this can be used as a treatment againstwitchcraft or as a treatment for a woman who has recently given birth)	0.40%	66%	38%
Asthma medicine	0.40%	19%	8%
Medicine for erectile dysfunction	0.37%	35%	20%
Medicine for period pains	0.37%	16%	15%
Medicine for chest pains	0.37%	25%	16%
Medicine for cramps, pains and chills after having given birth; also cleansingthe afterbirth	0.37%	85%	40%

Treatments that were infrequently used (subjectively categorized as less than 10 household-reports of a given treatment) are presented in Table 2. Particularly difficult to define and differentiate were a host of illnesses that relate to fatigue. *Ahinjanana* and *arerahana,* two regional names for the same illness, refer to fatigue that consumes the entire body and can either be physical or mental. *Fiandry* is a related illness that affects the kidneys and is characterized by fatigue and bright yellow and odorous urine. Two modes of illness for *fiandry* include labor overexertion leading to dehydration or the consumption of either overly sweet foods or too many medications. *Hozatra* and *gajogajo*, two regional names for the same illness, describe fatigue derived from extreme physical exertion. *Andilana* is a type of fatigue and muscle pain (centered at hips and lower back) that is caused by people who overexert during manual labor. *Andilana* could be considered a subset of *hozatra* but it is very commonly reported and often receives differing medications, thus obtaining its own category.

**Table 2:** The frequency of rarely-used botanical ethnomedicines.

Treatment Type	Percent	Preference for ethnomedicine(distant hospital)	Preference for ethnomedicine(on-site hospital)
Medicine for the spleen	5.26%	25%	30%
Medicine to cleanse the blood	5.26%	61%	29%
Medicine for exhaustion and shortness of breath	4.61%	12%	5%
Indigestion medicine	4.61%	5%	13%
Medicine for cramps	4.61%	34%	22%
Medicine for tetanus	4.61%	21%	7%
Medicine for the liver	3.95%	67%	12%
Medicine for the eyes	3.95%	27%	6%
Blood clotting medication	3.29%	43%	14%
Medicine for a type of disease that is not God-given or caused by bacteria butsent by an evil person	2.63%	100%	100%
Medication for genital discharge and burning urine	2.63%	20%	13%
Medicine for open cuts	2.63%	25%	14%
Medicine for enlarged testicles	2.63%	41%	25%
Flu medicine	2.63%	39%	10%
Medicine for intestinal worms/parasites	2.63%	39%	10%
Dehydration medicine	2.63%	65%	47%
Nausea medicine	1.97%	95%	44%
Sleep medicine	1.97%	0%	3%
Medicine for inflammation	1.97%	12%	7%
Medicine for lice/mites, etc.	1.97%	22%	6%
Medicine to induce labor contractions	1.97%	43%	15%
Medicine for hypertension	1.97%	69%	9%
Medicine to treat Tinea versicolor	1.32%	30%	15%
Medicine for the appendix	1.32%	24%	4%
Medicine for bloating and gaseousness	1.32%	78%	58%
Medicine for veins and arteries	1.32%	12%	7%
Blood thinner	1.32%	26%	7%
Medicine to stop vomiting	1.32%	5%	10%
Calcium supplement	1.32%	16%	7%
Medicine for yellow, painful eyes	1.32%	24%	16%
Anti-poison	1.32%	19%	7%
Medicine for foot pain	1.32%	23%	14%
Arthritis medicine	1.32%	44%	49%
Medicine for earaches	1.32%	19%	7%
Vitamin supplement (general)	1.32%	65%	50%
Medicine for dizziness or unclear vision	0.66%	31%	21%
Medicine to counter a cold body	0.66%	56%	16%
Medicine for rashes or itchy skin	0.66%	25%	25%
Medicine for rotten teeth in children	0.66%	69%	50%
Measles medicine	0.66%	6%	2%
Medicine for a headache (but specifically right above the eyes)	0.66%	10%	8%
Medicine to soften the stool	0.66%	58%	14%
Medicine to cleanse teeth	0.66%	38%	4%
Medicine for body swelling; often in reference to a hangover	0.66%	30%	10%
Medicine for a major system shock (e.g. after a major fall when there is swelling)	0.66%	79%	39%
Medicine for boils	0.66%	36%	8%
Medicine for tumefaction or swollen glands	0.66%	64%	10%
Medicine for sore throats	0.66%	32%	14%
Medicine following a miscarriage	0.66%	65%	13%
Medicine following birth	0.66%	27%	6%
Use jointly during a massage	0.66%	70%	43%
Medicine for hypertension	0.66%	29%	14%
Birth control (regulates period)	0.66%	25%	7%
Medicine for gonorrhea	0.66%	23%	5%

**Figure 2:**
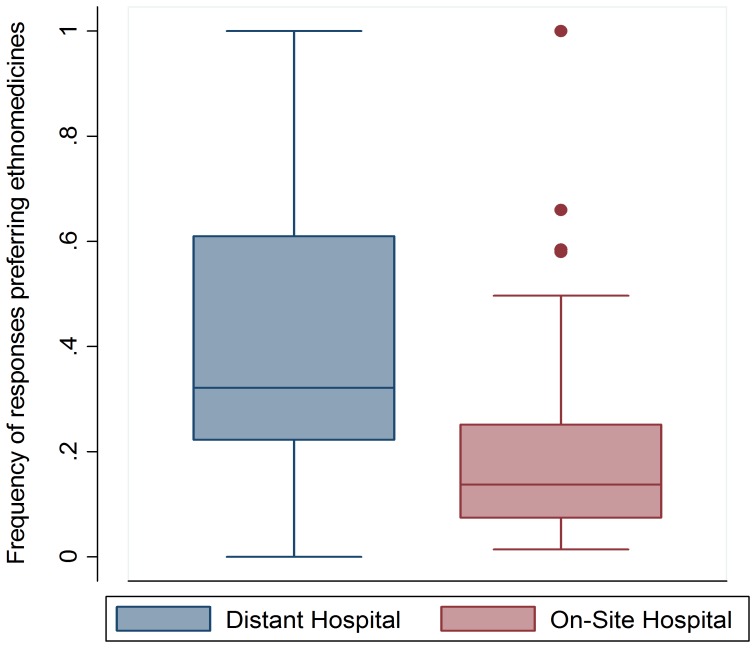
Preference for ethnomedicines affected by access to a hospital and pharmaceuticals. The presence of a hospital with an easily accessible pharmacy affects the preference of the perceived efficacy of ethnomedicines. Those respondents who lived in a village with a hospital and pharmacy were nearly three times (odds ratio = 2.7, p<0.0005) more likely to prefer pharmaceuticals to treat a given illness.

Averaged across all illness types, 38.1% (SD: 2.5%) of responses from inhabitants of villages without a hospital and pharmacy noted a preference for the ethnobotanical treatment, whereas 18.5% (SD: 1.7%) of responses from inhabitants who had access to a hospital and pharmacy still preferred an ethnobotanical treatment in terms of perceived efficacy (Fig. 2). Preference for the perceived efficacy of pharmaceuticals was found to be nearly three times higher for residents of villages with a hospital (odds ratio across illnesses clustered by individual = 2.7: z = 12.60, 95%CI: 2.3–3.2, p<0.0005, Fig. 2). Of the 140,000 individuals in the Makira Protected Area, approximately 8,120 (5.8% of residents) are likely to not use botanical ethnomedicines. Of the remaining individuals using botanical ethnomedicines (131,880), botanical ethnomedicines are accessed on average once per week (1.0±1.1). Through extrapolation of individual users and frequency of use, there are 6,878,333 individual-uses throughout the year in this area. By matching each illness with a Western medicine counterpart (when possible), the mean benefits of ethnomedicines per year was approximately $5.40–7.90 per person per year, $30.20–44.30 per household per year, and between $756,050–1,110,220 per year for all Makira residents (Table 3). The benefits from this provisioning service per household per year are equivalent to 43–63% of their median annual household liquid income and are obtained at low cost from the environment. If prices from American online drugstores are used, these medicines would be valued at $100.60–287.40 per person per year- approximately $14–40 million for all residents of the Makira Protected Area.

**Table 3:** The ecosystem-service provisioning value of botanical ethnomedicines.

Treatment Type	Substitute^1^	Adjusted Price (USD)	Value per year (USD)
Medicine for fatigue and muscle soreness	Ibuprofen	0.99–1.19	295,336–356,618
Medicine for fatigue and dehydration	Ampicillin	1.09–1.25	233,433–268,230
Medicine for lower back and hip pain	Ibuprofen	0.33–0.38	48,275–55,718
Stomach medicine	Chloramphenicol	0.37–1.16	47,637–148,861
Fever medicine	Chloroquine	0.01–0.04	540–2,492
Medicine for muscle fatigue	Vitamin B Complex	0.49–1.00	28,859–59,392
Medicine for back pain	Ibuprofen	0.21–0.34	11,853–19,433
Medicine to increase strength	Alvit	0.20–1.11	5,445–29,721
Malaria medicine	Actipal	0.33–0.69	8,555–18,105
Medicine for enlarged testicles (also can mean a medicine to cure bedwetting)	Ampicillin	0.77–1.33	10,538–18,099
Medicine for headaches	Acetaminophen	0.01	149
Medicine for anemia	Astyfer	0.58–1.38	7,049–16,837
Medicine for gastro-intestinal ailments	Cimetidine	0.37–0.56	4,065–6,100
Medicine for dizziness or vertigo	Calcium gluconate	0.41–0.83	4,133–8,241
Medicine to cure tiredness	Calcium	0.32–1.02	2,873–9,214
Medicine for painful wisdom teeth	Nifluril	0.05–0.28	461–2,409
Cough medicine	Cotrim	0.05	438
Medicine to treat jaundice	Furosemide	0.14–0.38	989–2,720
Medicine for toothaches	Dicofenac	0.06–0.17	404–1,171
Diarrhea medicine	Chloramphenicol	0.24–0.64	1,414–3,776
Medicine to treat genital sores and ulcers	Genicure	0.03–0.16	174–890
Medicine for warming the stomach (this can be used as a treatment against witchcraft or as a treatment for a woman who has recently given birth)	Clomid	2.30–3.97	11,492–19,779
Asthma medicine	Theophylline	0.14–0.33	701–1,627
Medicine for erectile dysfunction	Vitamin B Complex	0.41–0.70	1,843–3,182
Medicine for period pains	Kenacort	0.91–0.97	4,110–4,387
Medicine for chest pains	Ibuprofen	0.11–0.17	484–753
Medicine for cramps, pains and chills after having given birth; also cleansingthe afterbirth	Metronidazole/ Amoxicylline	0.57–1.22	2,571–5,524
Medicine for the spleen	Diclofenac	0.22	806
Medicine to cleanse the blood	Benzathine	1.15–2.44	4,160–8,852
Medicine for exhaustion and shortness of breath	Theophylline	0.09–0.21	284–670
Indigestion medicine	Cimetidine	0.27	846
Medicine for cramps	Ibuprofen	0.22–0.39	785–1,229
Medicine for tetanus	Bipenicylline	0.39–1.24	1,242–3,940
Medicine for the liver	Furosemide	0.15–0.84	416–2,286
Medicine for the eyes	Tetracycline	0.04–0.17	114–472
Blood clotting medication	Dicynone	0.23–0.71	519–1,612
Medicine for a disease that is not God-given or caused by bacteria butsent by an evil person	No Treatment		
Medication for genital discharge and burning urine	Cura7	0.08–0.12	141–214
Medicine for open cuts	Bipenicylline	0.42–0.76	761–1,383
Medicine for enlarged testicles	Ampicillin	0.57–0.93	1,039–1,678
Flu medicine	Efferalgan	0.09–0.35	160–632
Medicine for intestinal worms/parasites	Mebendazol	0.07–0.28	128–509
Dehydration medicine	Tres-Orix	2.85–3.89	5,166–7,063
Nausea medicine	Mebendazol	0.37–0.81	507–1,098
Sleep medicine	Diazepam	0.04	58
Medicine for inflammation	Ampicillin	0.12–0.20	158–271
Medicine for lice/mites, etc.	Gentamicine	0.08–0.28	106–378
Medicine to induce labor contractions	Benzanthine	0.59–1.71	796–2,332
Medicine for hypertension	Hept-A-Myl	0.18–1.37	242–1,867
Medicine to treat Tinea versicolor	Miconazole	0.23–0.45	207–408
Medicine for the appendix	Chloramphenicol	0.07–0.50	67–454
Medicine for bloating and gaseousness	Active Charcoal	0.29–0.39	265–353
Medicine for veins and arteries	Aspirin	0.02–0.03	17–27
Enhance coagulation	Vitamin K1	0.28–0.98	254–884
Medicine to stop vomiting	Metoclopramide	0.06	52
Calcium supplement	Calcibronat	0.26–0.62	238–563
Medicine for yellow, painful eyes	Furosemide	0.18–0.26	159–240
Anti-poison	Cimetidine	0.09–0.23	80–210
Medicine for foot pain	Ibuprofen	0.07–0.11	63–104
Arthritis medicine	Ibuprofen	0.24	220
Medicine for earaches	Tetracycline	0.05–0.13	41–122
Vitamin supplement (general)	Ananambo	0.02–0.03	23–29
Medicine for dizziness or unclear vision	Calcium	0.31–0.46	141–209
Medicine to counter a cold body	Quinine	0.12–0.43	54–194
Medicine for rashes or itchy skin	Ampicillin	0.48–0.49	218–222
Medicine for rotten teeth in children	Fungizone	1.12–1.56	506–707
Measles medicine	Ampicillin	0.04–0.12	20–55
Medicine for a headache (but specifically right above the eyes)	Ibuprofen	0.04–0.05	19–22
Medicine to soften the stool	Forlax	0.25–1.05	112–476
Treatment Type	Substitute^1^	Adjusted Price (USD)	Value per year (USD)
Medicine to cleanse teeth	Teeth cleaning	0.13–1.13	59–513
Medicine for body swelling; often in reference to a hangover	Benzylpenicillin	0.38–1.13	172–513
Medicine for a major system shock (e.g. after a major fall when there is swelling)	Diclofenac	0.22–0.43	98–197
Medicine for boils	Ampicillin	0.15–0.70	70–317
Medicine for tumefaction or swollen glands	Amoxicillin	0.22–1.34	98–609
Medicine for sore throats	Amoxicillin	0.21–0.48	97–219
Medicine following a miscarriage	Ampicillin	0.28–1.36	125–618
Medicine following birth	Vitamine K1	0.22–1.00	101–455
Use jointly during a massage	Diclofenac	0.22–0.35	98–159
Medicine for hypertension	Furosemide	0.17–0.36	79–162
Birth control (regulates period)	Confience	0.03–0.12	14–55
Medicine for gonorrhea	Ciprofloxacin	0.05–0.24	25–109

1 Substitutes listed here are those recommended by a physician in Maroantsetra and are not necessarily the pharmaceutical that should be prescribed.

There is a median of 48.5 households per village (IQR: 16–112.5) and, using the midpoint of the range of values per household, a median benefit (*B*) of $16.89/ha/yr (IQR: 5.57–39.19). The median time traveled to access botanical ethnomedicines was 7 minutes, the equivalent of approximately 0.58 km of linear distance and an average harvest area of 1.07 km^2^. Including approximately 5 minutes for harvesting, the round trip time allocation was approximately 19 minutes. The costs of allocating this time were approximately $0.55 per household per year and $0.13 per hectare per year. This produces a median net value of $16.76/ha/yr (IQR: 5.44–39.06).

Notably absent from botanical ethnomedicines were any use of preventative medicines. Although not reported from botanicals, there are other types of ethnomedicines (i.e. soils) that are used as preventative medicine, locally called *aody fiarovana*. The local Malagasy have a very advanced conception of disease and use the metaphor of the word fence (*fefiny*) to convey the action of this type of medicine. Interestingly absent from all ethnomedical treatments were any types of psychiatric remedies for diseases such as depression, schizophrenia, etc. This is not to say that certain ethnomedicines do not provide support for mental health writ large (i.e. fatigue, witchcraft protection, etc.). For other types of medicine, there is no applicable Western biomedical treatment. For instance, *aody aretina miforona* refers to diseases that are sent through curses and witchcraft. In addition to ethnobotanical medicine, local Malagasy also used forms of ethnozoological medicine. Both the throat meat of the black and white ruffed lemur, *Varecia variegata*, and raw blood from the common tenrec, *Tenrec ecaudatus*, are used to treat pertussis. The fat from the fosa, *Cryptoprocta ferox*, is boiled down to an oil and applied as a cream to treat earaches. Similarly, the fat from Nile crocodile, *Crocodylus niloticus*, meat is used as a general curative to treat cancer and a variety of other ailments. The vagina from the zebu, *Bos indicus*, is cut from the animal and put into water and squeezed of all of its juices. This raw concoction is then consumed to treat childhood asthma. The raw liquid contents of a zebu’s gallbladder are consumed just after the zebu’s death also to treat asthma. Because the forest also contains soils and animals that are used as medicine, this valuation is only a conservative estimate of the ethnomedical value of tropical forests in this region.

## Discussion

The Malagasy repertoire for botanical ethnomedicinal treatment is well- developed and complex. There are 82 categories of illness identified that can be treated by 241 locally-recognized plant species; for comparison, trained physicians employ 47 different types of pharmaceuticals to treat the same illnesses in the region. Local Malagasy perceive that treatments have improved efficacy when they are tailored to the individual. Thus, the diversity and specificity of ethnomedicines “designed” to treat a specific illness increases the perceived value. Additionally, many treatments are gender-specific, also heightening their perceived value. The local Malagasy of course value this service, although not monetarily, and it is a clear example of what Pattanayak and Sills [13] have considered “natural insurance,” where forest resources serve as a buffer against shocks or provide a service that is prohibitively expensive to use otherwise. We estimated a net value of $17 per hectare per year, very similar to estimates from Central America [14]. We demonstrate that the value of ethnomedicines could be $30–45 per household per year, equivalent to 43–63% of the median annual household income. This income measure is the value of all products sold, wages earned and items bartered but does not include liquid assets such as agriculture, etc. Using the price of American pharmaceuticals as a substitute, individuals receive approximately $100–290 per year. This is approximately 22–63% of the median annual health care expenditure for American adults under 45 years of age in 2006 [15]. Local people also place an existence value on the fact that they can rely on this provisioning service with such immediacy. Even households not well-versed in ethnomedicine use can rely on neighboring households to collect for them, given the easy geographical access and strong kin networks. This provisioning service is not only valuable in its contribution to local Malagasy well-being but also for its potential to contribute to global knowledge and medical development.

There are several weaknesses in this study causing uncertainty in estimates. First, we are not explicitly accounting for the ecology of the ethnomedical plant production flows or how this production may be changed due to policy or human behavior [16].This valuation method may be an overestimate as it is premised on the exchangeability of ethnomedicines and pharmaceuticals. We tried to control for this by weighting values by perceived efficacy and preference but the true effectiveness of all of the botanical ethnomedicines in this study is unknown. These estimates also assume that there is an equivalent market demand for these medicines if the prices were equated to pharmaceuticals. A conservative estimate for the value of these medicines could be the calculations of time allocation costs from Equation 2. The time allocated to collecting these medicines would be the opportunity cost of not participating in another income gaining activity or the shadow value of these ethnobotanicals. What may be less obvious are the reasons why the calculation here may be an underestimate of its true value. First, there are no Western medicines for certain ailments that are recognized by Malagasy people (e.g. illnesses transferred by curse or witchcraft). Secondly, ethnomedicines could be deemed as a safer alternative to pharmaceuticals. Most botanical ethnomedicines have never been tested but we can assume their safety and efficacy due to long-standing historical use [17], [18]. Moreover, there can be major negative effects of prescribing overly potent pharmaceutical treatments or mistreating infections with improper medications, particularly in Madagascar. Lack of adequate medical training, dumping of expired pharmaceuticals from France and the ability for non-professionals to “prescribe” and sell pharmaceuticals all complicate the safety and performance of Western medicines. For instance, diazepam is the pharmaceutical used to treat sleeplessness in Madagascar. Diazepam can cause retrograde amnesia and is likely to cause physical dependence [19]. Similarly, fungizone is used to treat rotten teeth in children in the study area and yet this medication is typically reserved in the West only for progressive and life-threatening fungal infections [20]. Side effects of these strong pharmaceuticals are often left medically unmanaged as local people return to their village after receiving medication. Antibiotics are being prescribed for non-bacterial infections such as the use of Cotrim to treat coughs when it is likely that the majority of throat infections are viral and not bacterial [21], [22]. This raises issues of antibiotic resistance from overusing antibiotics in this system. There are also potentially significant interactions between ethnomedicines and pharmaceuticals that could be dangerous or inhibit pharmaceutical efficacy [23], [24].

The benefit calculation in this study is derived from the valuation of alternative treatments available, taking time allocation costs into account but not considering the previously-listed drawbacks of pharmaceuticals. Moreover, the calculation does not include a value for the profitability of a new drug discovered from this rainforest. It has been estimated that the Makira watershed area contains 25% of Malagasy botanical biodiversity [25]. There are 12,000 species of plants in Madagascar, approximately 80% of which are endemic, comprising 3.2% of global plant biodiversity [2]. This would mean that this one watershed area comprises approximately 0.8% of total global botanical diversity. According to Mendelsohn and Balick [26], for each species of plant, there are three different parts, two different extraction procedures and 500 screens per sample, which in this study, would yield 1,836 extracts and require 918,000 tests. Of these tests, approximately one in 50,000 to one in a million tests may produce an effective and viable commercial pharmaceutical drug [26], [27]. Employing this success rate to the number of possible tests for this study system, we may assume that there are between 1–18 potentially novel drugs in the Makira Protected Area. Using the average sales value of novel FDA-approved pharmaceuticals between 2000–2010 [28], each new botanically-based pharmaceutical is worth $316 million in revenue in the USA (without subtracting the costs of research and development, among others). This could mean that the MPA holds between $316 million to almost $6 billion of untapped revenue within its botanical diversity.

The Reducing Emissions from Deforestation and Degradation (REDD+) program is a mechanism to increase carbon sequestration and negotiate value of intact forests in a market setting [29]. In the Makira Protected Area (a REDD+ forest), an expected 9.2 million metric tons of carbon were sequestered through the protection of the forest and the halting of deforestation patterns [30]. The current sales price for carbon credits is $3 per metric ton, equaling approximately $27.6 million for the entire MPA, as compared to the $14–40 million accrued by residents annually from ethnomedicines if we use the US pharmaceutical price equivalents. On a per hectare basis, Winrock International,the firm who assessed carbon stocks in the MPA, calculated that there was approximately 1,000 metric tons of carbon dioxide (app. 272.5 metric tons of carbon) in each hectare of forest [31]. The value of the forest for REDD per hectare ($818) pales in comparison to its ethnomedicinal value to local people ($17/ha using US pharmaceutical prices) in addition to its potential value as a source of pharmaceuticals for society.

However, REDD+ values or royalties from the discovery of new medicines would not be as meaningful to local people, because they would be unlikely to be equitably distributed to the majority of people in the region. Although the net value of ethnomedicines is wholly obtained by local people, only a very small percentage of pharmaceutical or REDD benefits may actually trickle down to Makira residents. Certain important medicines may become more scarce and difficult to access as deforestation unolds in this area. However, almost 50% of the botanical ethnomedicines are agricultural or weed species that may not be affected by deforestation and habitat loss. In fact, the cultural and health values of ethnomedicines may be additional to the forests value as a source of carbon sequestration if these medicines are harvested sustainably. Thus, this study does not encounter the same political context of restricted use that arrives from conservation enforcement (i.e. [32], [33]). It is possible that none of these values are mutually exclusive and that the value of this forest will provide protection of critically endangered biodiversity, intellectual and medicinal inspiration, religious and health value through local ethnobotany, and buffering against the effects of future climate change through carbon sequestration.

## Supporting Information

Table S1 **Treatment names in Malagasy.** These are the translations of the treatment names in the local dialect of the Maroantsetra region, characterized by the Betsimisaraka and Tsimihety ethnic groups.
(DOCX)

Table S2 **Regional and dialectical variants in illness identification.** The Makira Protected Area is a large region that spans many regional districts where the Betsimisaraka and Tsimihety live. Even within this one region, there are dialectical nuances to describe what the authors believe to be the same illness.
(DOCX)
