# Supplementary material for: Rainforest Pharmacopeia in Madagascar Provides High Value for Current Local and Prospective Global Uses
Source: PLoS One. 2012 Jul 27;7(7):e41221. doi: 10.1371/journal.pone.0041221 (PMC3407148; doi:10.1371/journal.pone.0041221)
Supplement: Table S1 — Treatment names in Malagasy. These are the translations of the treatment names in the local dialect of the Maroantsetra region, characterized by the Betsimisaraka and Tsimihety ethnic groups. (DOCX) [file pone.0041221.s001.docx]

| **Treatment Type** | **Malagasy Translation** |
| --- | --- |
| Medicine for physical and mental fatigue | Aody ahinjanana |
| Medicine for fatigue and dehydration | Aody fiandry |
| Medicine for lower back and hip pain | Aody andilana |
| Stomach medicine | Aody kibo |
| Fever medicine | Aody tazo |
| Medicine for muscle fatigue | Aody hozatra |
| Medicine for back pain | Aody tahaizana |
| Medicine to increase strength | Aody mampatanjaka |
| Malaria medicine | Aody tazo mahery |
| Medicine for enlarged testicles  (also can mean a medicine to cure bedwetting) | Aody sorondrano |
| Medicine for headaches | Aody loha |
| Medicine for anemia | Aody tsy ampy ra |
| Medicine for gastro-intestinal ailments | Aody vavony |
| Medicine for dizziness or vertigo | Aody fanimaso |
| Medicine for painful wisdom teeth | Aody vazana |
| Cough medicine | Aody satra |
| Medicine to treat jaundice | Aody angorohosy |
| Medicine for toothaches | Aody nify |
| Diarrhea medicine | Aody fivalanana |
| Medicine to treat genital sores and ulcers | Aody farasisa |
| Medicine for warming the stomach (this can be used as a treatment against witchcraft or as a treatment for a woman who has recently given birth) | Aody mampafana votraka |
| Asthma medicine | Aody sohiky |
| Medicine for erectile dysfunction | Aody fanenjana |
| Medicine for period pains | Aody marary fotoana |
| Medicine for chest pains | Aody tratra |
| Medicine for cramps, pains and chills after having given birth; also cleansing the afterbirth | Aody lalavy |
| Medicine for the spleen | Aody katry |
| Medicine to cleanse the blood | Aody manala ra maloto |
| Medicine for exhaustion and shortness of breath | Aody sesika |
| Indigestion medicine | Aody tarafo |
| Medicine for cramps | Aody hotsokotsoko |
| Medicine for tetanus | Aody tetanosy |
| Medicine for the liver | Aody aty |
| Medicine for the eyes | Aody maso |
| Blood clotting medication | Aody mampijanona ra |
| Medicine for a type of disease that is not God-given or caused by bacteria but sent by an evil person | Aody aretina miforona |
| Medication for genital discharge and burning urine | Aody solopiso |
| **Treatment Type** | **Malagasy Translation** |
| Medicine for open cuts | Aody fery |
| Medicine for enlarged testicles | Aody hangalahy |
| Flu medicine | Aody rehoreho |
| Medicine for intestinal worms/parasites | Aody viky |
| Dehydration medicine | Aody manala hetaheta |
| Nausea medicine | Aody hambo |
| Sleep medicine | Aody manome toromaso |
| Medicine for inflammation | Aody mivonto |
| Medicine for lice/mites, etc. | Aody pia |
| Medicine to induce labor contractions | Aody sahana |
| Medicine for hypertension | Aody tension |
| Medicine to treat Tinea versicolor | Aody bolia |
| Medicine for the appendix | Aody apandasity |
| Medicine for bloating and gaseousness | Aody arakaraka |
| Medicine for veins and arteries | Aody lalandra |
| Blood thinner | Aody mampihena ra |
| Medicine to stop vomiting | Aody mampijanona mandoa |
| Calcium supplement | Aody manampy calcium |
| Medicine for yellow, painful eyes | Aody maso fondrana |
| Anti-poison | Aody poison |
| Medicine for foot pain | Aody tongotra |
| Arthritis medicine | Aody vadikozatra |
| Medicine for earaches | Aody tadigny |
| Vitamin supplement (general) | Aody vitamine gasy |
| Medicine for dizziness or unclear vision | Aody fanompanomaso |
| Medicine to counter a cold body | Aody hatsiaka |
| Medicine for rashes or itchy skin | Aody hoditra mangidigidy |
| Medicine for rotten teeth in children | Aody kady |
| Measles medicine | Aody kisosy |
| Medicine for a headache (specifically right above the eyes) | Aody loha (vajihy) |
| Medicine to soften the stool | Aody mampalemy tay |
| Medicine to cleanse teeth | Aody manadio nify |
| Medicine for body swelling, often in reference to a hangover | Aody mibobohaka |
| Medicine for a major system shock (e.g. after a major fall when there is swelling, etc.) | Aody mihaitry |
| Medicine for boils | Aody mosindry |
| Medicine for tumefaction or swollen glands | Aody tangiky |
| Medicine for sore throats | Aody tendana |
| Medicine following a miscarriage | Aody zaza niala |
| Medicine following birth | Aody zaza vao teraka |
| Use jointly during a massage | Fanotrorana |
| Medicine for hypertension | Mampihena tosi-dra |
| Birth control (regulates period) | Manampim-bolana |
| Medicine for gonorrhea | Aody blenoragie |
