# Supplementary material for: Rainforest Pharmacopeia in Madagascar Provides High Value for Current Local and Prospective Global Uses
Source: PLoS One. 2012 Jul 27;7(7):e41221. doi: 10.1371/journal.pone.0041221 (PMC3407148; doi:10.1371/journal.pone.0041221)
Supplement: Table S2 — Regional and dialectical variants in illness identification. The Makira Protected Area is a large region that spans many regional districts where the Betsimisaraka and Tsimihety live. Even within this one region, there are dialectical nuances to describe what the authors believe to be the same illness. (DOCX) [file pone.0041221.s002.docx]

| **Most commonly used term** | **Regional variations in dialect** |
| --- | --- |
| Aody ahinjanana | Aody arerahana |
| Aody aretina miforona | Aody fanafana mosavy, aody gasy |
| Aody kibo | Aody boko, aody aretim-botraka, aody votraka |
| Aody andilana | Aody vaniagna |
| Aody tadigny | Aody sofina |
| Aody tsy ampy ra | Aody fanampindra |
| Aody rehoreho | Aody sery |
| Aody hozatra | Aody gajogajo |
